# Rodent behavior following a dural inflammation model with anti-CGRP migraine medication treatment

**DOI:** 10.3389/fneur.2023.1082176

**Published:** 2023-02-23

**Authors:** Philip V. Reducha, Jesper P. Bömers, Lars Edvinsson, Kristian A. Haanes

**Affiliations:** ^1^Department of Clinical Experimental Research, Glostrup Research Institute, Copenhagen University Hospital, Glostrup, Denmark; ^2^Section of Cell Biology and Physiology, Department of Biology, University of Copenhagen, Copenhagen, Denmark; ^3^Department of Neurosurgery, Copenhagen University Hospital–Rigshospitalet, Copenhagen, Denmark; ^4^Division of Experimental Vascular Research, Department of Clinical Sciences, Lund University Hospital, Lund, Sweden

**Keywords:** inflammation, migraine, calcitonin gene-related peptide (CGRP), complete Freund's adjuvant (CFA), inflammatory soup

## Abstract

**Background:**

Migraine is a widespread and prevalent disease with a complex pathophysiology, of which neuroinflammation and increased pain sensitivity have been suggested to be involved. Various studies have investigated the presence of different inflammatory markers in migraineurs and investigated the role of inflammation in inflammatory models with complete Freund's adjuvant (CFA) or inflammatory soup added to the dura mater.

**Objective:**

The aim of the current study was to examine whether application of CFA to the dura mater would cause behavioral alterations that are migraine relevant. In addition, we investigated the potential mitigating effects of fremanezumab, a CGRP (calcitonin gene-related peptide) specific antibody, following CFA application.

**Methods:**

Male Sprague-Dawley rats were randomly divided into six groups: fresh (*n* = 7), fresh + carprofen (*n* = 6), fresh + anti-CGRP (*n* = 6), sham (*n* = 7), CFA (*n* = 16), CFA + anti-CGRP (*n* = 8). CFA was applied for 15 min on a 3 × 3 mm clearing of the skull exposing the dura mater of male Sprague-Dawley rats. We applied the Light/Dark box and Open Field test, combined with the electronic von Frey test to evaluate outcomes. Finally, we observed CGRP immunoreactivity in the trigeminal ganglion.

**Results:**

No differences were observed in the Light/Dark box test. The Open Field test detected behavior differences, notably that sham rats spend less time in the central zone, reared less and groomed more than fresh + carprofen rats. The other groups were not significantly different compared to sham rats, indicating that activation of the TGVS is present in sham surgery and cannot be exacerbated by CFA. However, for the allodynia, we observed specific periorbital sensitization, not observed in the sham animals. This could not be mitigated by fremanezumab, although it clearly reduced the amount of CGRP positive fibers.

**Conclusion:**

CFA surgically administered to the dura causes periorbital allodynia and increases CGRP positive fibers in the trigeminal ganglion. Fremanezumab does not reduce periorbital allodynia even though it reduces CGRP positive fibers in the TG. Further work is needed to investigate whether CFA administered to the dura could be used as a non-CGRP inflammatory migraine model.

## 1. Introduction

Migraine is a widespread and prevalent disease with a complex pathophysiology, of which neurogenic neuroinflammation (1) and increased pain sensitivity (2) have been suggested to be involved. Neurogenic neuroinflammation is defined as inflammatory reactions occurring in the central nervous system and peripheral nervous system triggered by enhanced neuronal activity. In the context of migraines, it refers to the terminal release of peripheral vasoactive substances–such as calcitonin gene-related peptide (CGRP) and substance P–from sensory neurons and fibers of the trigeminovascular system (TGVS) (3, 4). The release of vasoactive peptides can create various consequences in the TGVS including vasodilation of surrounding meningeal and cranial arteries (5), plasma extravasation (6), mast cell degranulation (7), release of pro-inflammatory cytokines (8), chemokines and mediators (9) and lastly nociceptive signaling (10), leading to the generation of pain (11). In patients increased CGRP levels and release have been observed during migraine attacks, which can last up to 72 h. A prolonged and continuous release cycle is suggested to cause peripheral/central sensitization and eventually the chronification of migraines (12).

The impact of neuroinflammation in migraine is yet to be defined. Various studies have investigated the presence of different inflammatory markers. TNF-α and C-reactive proteins levels were reported to be increased in both episodic and chronic migraineurs (13). Peripheral blood levels of NGF, BDNF, PGE2, and VEGF have been reported to be increased in migraine patients as well (14). Furthermore, in a study conducted by Yucel et al. (15), serum levels of IL-1β, IL-6 and TNF-α levels were higher in migraineurs compared to controls and were elevated further during migraine attacks. In term of treatments, using TNF-α antibodies did not alleviate symptoms in migraine patients and arguably triggered migraines (16, 17), challenging the view of inflammation as an initiator of debilitating migraine symptoms. However, some treatments that alleviate migraines symptoms in addition to having anti-inflammatory effects have been reported. For example, supplementation with ω-3 fatty acids and nano-curcumin, reduced expression, and serum levels of TNF-α in addition to reducing migraine attack frequency (18). However, whether the anti-inflammatory effects played a big part in the alleviating effects or if it was only a side effect of the treatments remains unclear. The same question can be applied to the effectiveness of known anti-migraine drugs, like nonsteroidal anti-inflammatory drugs or triptans (19). These drugs have anti-inflammatory properties but whether the anti-inflammatory effects are one of the drivers of the migraine alleviating effects remains unclear, as they also possess other properties.

While the discussion on the involvement of inflammation in migraine pathophysiology is continued, many inflammation and pain sensitization animal models have been developed. Models like these help shape our understandings of migraines, identifying potential biomarkers and aid to identify or create new migraine treatments. Evaluating migraine relevant behavior and biomarkers in animal models of inflammation can help us to deduce whether these models are translatable. To stimulate inflammation, the two most commonly applied inflammatory stimulants are complete Freund's adjuvant (CFA) or inflammatory soup (IS). CFA (dried inactivated Myobacterium tuberculosis in mineral oil) stimulates an immune response causing inflammatory reactions while IS (mixture of serotonin, histamine, PGE2 and bradykinin) consists of endogenous factors released during inflammation thereby developing an inflammatory state and creating a “sterile inflammation”. In the study applying CFA to the dura mater (20), both aforementioned inflammatory mediators led to the activation of the trigeminal ganglion (TG), potentially due to neuro-glial interaction, as increased expression of pERK1/2 in satellite glial cells and IL-1β in neuronal cytoplasm was observed. The expression levels of CGRP, the highly relevant migraine neuropeptide, was also increased in neurons and nerve fibers of the TG (20). It has been reported using CFA in pain models, like trigeminal neuralgia models or temporomandibular joint models, successfully activate the TGVS (1, 21). CFA applied to the facial areas cause upregulation of inflammatory markers in the TGVS and can provoke behaviors that are also migraine relevant (22–24). While we found that CFA applied to the dura activate the TGVS, its validity as a model would further be strengthened if it also caused migraine-like behavior, which we set out to investigate in this study.

Treatments targeting CGRP or its receptor, such as gepants, CGRP monoclonal antibodies (mAbs) or triptans/ditans [which reduces CGRP as part of their mechanism (25)], are effective therapies for many migraineurs (26). Some triptans were shown effective at diminishing the pathological effects caused by IS (27, 28) or CFA (29, 30) in rodents. Inconsistent efficacy has been reported from studies using gepants (31–33), while CGRP mAbs have not yet been investigated as the experimental focus has been on the vasculature (34, 35). Investigating the effects of mAbs in inflammation models could help elucidate the role of inflammation in migraines, or whether CGRP is an important driver of inflammation. In addition, it is possible that some of the migraine pathology might have similarities with other diseases of the TGVS, such as trigeminal neuralgia, which has been reported to not respond to anti-CGRP treatment (36).

Various inflammation studies based on the protocol from Oshinsky et al. (21, 37) have been conducted where IS (or other chemical stimulants) are episodically applied to the dura of animals to mimic episodic or chronic migraine. From these studies, besides observing upregulation of various inflammatory markers, they also observed migraine-like behaviors. Of note, rats in the study conducted by Zhang et al. (38) felt more periorbital and hindpaw pain, indicating higher sensitivity to touch and potentially experienced headaches. Melo-Carrillo et al. (39) noted decreased locomotor activity and increased resting behavior in their rats following an inflammation stimuli, which are behaviors that are similarly observed in migraine patients. To date, similar investigations have not been conducted for CFA application. The aim of this study was therefore to test whether application of CFA to the dura mater would cause behavioral alterations that are migraine relevant. In addition, we investigated the potential mitigating effects of fremanezumab, a CGRP specific antibody, following CFA application.

## 2. Methods

### 2.1. Experimental design

Male Sprague-Dawley rats (250–400 g) were purchased from Taconic (Denmark). The animals were housed in Eurostandard cages (Type VI with 123-Lid) in groups of 3–6 animals. Rats were given food and water *ad libitum*, lived under constant temperature (+22°C) and humidity (55%) and were sustained at a 12/12-h light-dark cycle. The study followed the guidelines of the European Communities Council (86/609/ECC) and the procedure is approved by the Danish Animal Experimentation Expectorate, no. 2020-15-0201-00751.

A total of 50 rats were used and randomly divided into six groups: naïve rats (referred to as fresh, *n* = 6), naive rats that received two doses of 0.1 mL/100 g of 5 mg/mL carprofen similar to the animals where we performed the surgery (referred to as fresh + carprofen, *n* = 7), naive rats that had only received intravenous (IV) Fremanezumab (AJOVY, Teva Pharmaceuticals) (referred to as fresh + anti-CGRP, *n* = 6), rats who had surgery and CFA applied to the dura mater (referred to as CFA rats, *n* = 16), CFA rats treated with IV Fremanezumab (AJOVY, Teva Pharmaceuticals) (referred to as CFA + anti-CGRP, *n* = 8) and sham rats who underwent the same surgery except the application of CFA (referred to as sham, *n* = 7).

### 2.2. Surgery and injections

All animals were habituated for a minimum of 7 days before inclusion. On the day of the “surgery” (day 0) we infused anti-CGRP treatment in two of the groups: fresh + anti-CGRP and CFA + anti-CGRP. The dose of fremanezumab 30 mg/kg was chosen based on the acute experiments by Melo-Carrillo et al. (40) and the long-term experiment by Dux et al. (41). Fremanezumab was purchased from the local pharmacy (AJOVY, Teva Pharmaceuticals and diluted 1:5 in saline, before the IV injection). Fremanezumab efficiently binds rat CGRP (42).

To induce inflammation, we used a modified version of a model by Lukacs and colleagues (20). The animal was placed in an anesthesia induction chamber containing 5% isoflurane gas bell before being transferred and fixated on to a stereotaxic frame with built-in mask, maintaining anesthesia with 2–3% isoflurane gas. The fur on the scalp was removed and the area cleaned with chlorhexidine-ethanol. Local anesthetic, lidocaine with adrenaline, was applied subcutaneously at the midline. A midline incision was performed, and the bone was cleared of connective tissue using a cotton swab as a rougine. An Alm retractor was placed. 5 mm anterolateral to the bregma on the right side, a power drill made a 3 × 3mm clearing of the skull exposing the dura mater. 10 μl of Complete Freund's adjuvant (CFA, F5881, Merck, Germany) was applied in the burr hole, to the dura mater and left for 15 min. CFA (F5881) is a liquid where each mL contains 1 mg of Mycobacterium tuberculosis (H37Ra, ATCC 25177), heat killed and dried, 0.85 mL paraffin oil and 0.15 mL mannide monooleate. Afterwards, the indentation was filled with bone wax and the incision was closed using 4-0 nylon suture. The animal was transferred to a single-house cage before being reintroduced to its cage mates, ~4 h after surgery. The animal received 0.1 mL/100 g of 5 mg/mL carprofen subcutaneously perioperative and 24 h after surgery.

### 2.3. Behavioral tests

**Figure 1:**
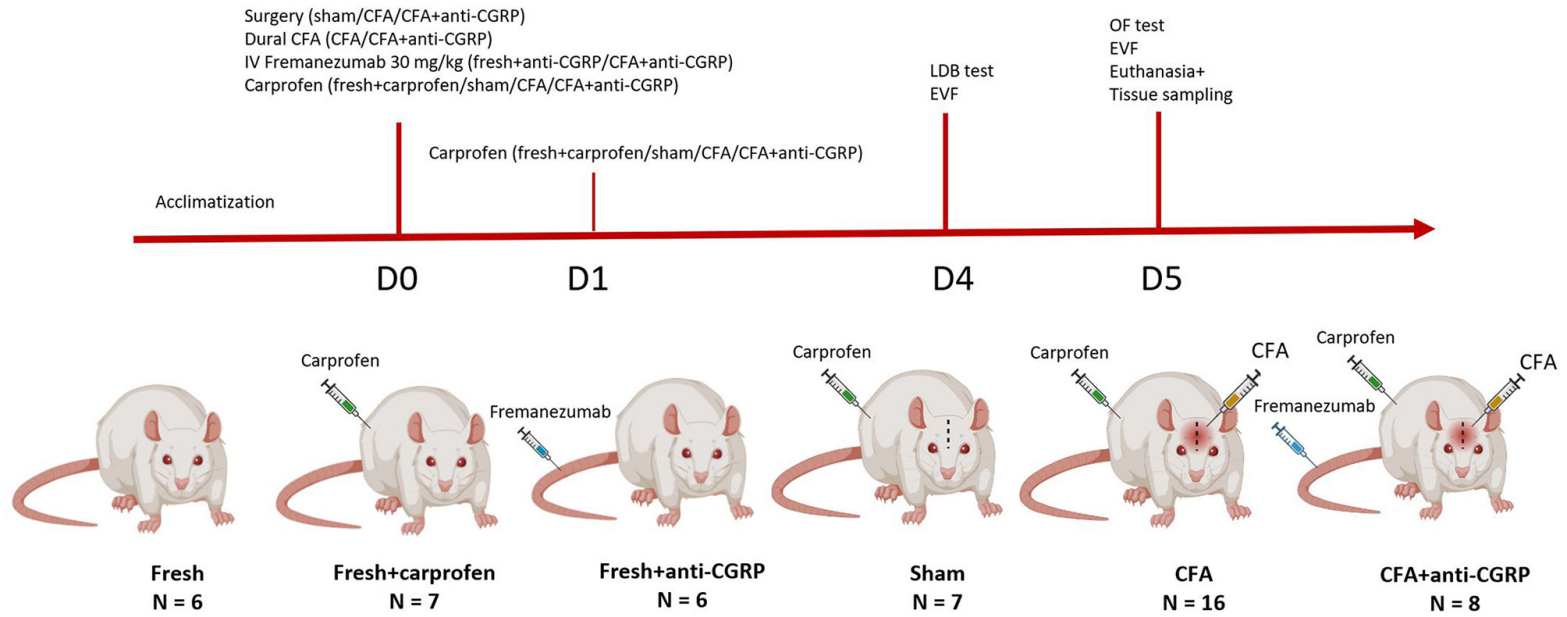
Experimental design. Illustrative depiction of the experimental timeline.

The behavioral study consisted of three tests: a Light/Dark box (LDB) test, an Open Field (OF) test and an electronical von Frey (EVF) test. LDB test and the OF test were performed between 10 and 12 a.m. EVF was performed between 01 and 04 p.m. On day 4 post-surgery, all rats underwent the LDB test and later the EVF test. On day 5, all rats underwent the OF test and later the EVF test. After the EVF test on day 5, all rats were euthanized, and their TG sampled for immunohistochemistry (IHC) (Figure 1).

#### 2.3.1. Light/dark box test

The LDB test allow to monitor photophobic behavior which is common behavior seen in migraine patients during attacks (43). Rats were individually tested in the LDB test on day 4 post-surgery. Rats in their cages were placed in the testing room around 9:30 a.m. for acclimatization, and testing were held between 10 and 12 a.m.

The setup consisted of two plexiglas boxes of identical size (50 × 50 cm and 34 cm in height). The light box had no lid and a light intensity of ~1,000 lux. This specific light intensity was chosen to stimulate a lighter environment, that would discourage rats that experience photophobia, while not discouraging healthy rats to explore. The second box consisted of darker walls and a top to make it pitch-dark, with plexiglass permeable to infra-red (IR) light, making video-recordings possible. A darker environment is believed to be more suitable for rats experiencing photophobia. There was an opening of 10 × 10 cm between the two boxes to facilitate transition between the boxes for the rats.

Rats were taken individually out of their cages and placed in the center of the light box with their heads facing away from the dark box. Rats were recorded and tracked using the ANY-MAZE (Stoetling, USA) software and an IR lamp to monitor their behavior. Time spent in the light zone (seconds) was chosen as parameter for analysis of photophobic behavior. Distance traveled per minute spent in the dark box was a parameter chosen for the reduced physical activity migraineurs experience during migraine attacks. The individual rats were monitored for 10 min. When testing was completed for each rat, both boxes were cleaned with 20% ethanol to remove scent of previous rats to avoid discrepancies and distractions between tests. The investigator was blinded to the group designation.

#### 2.3.2. Open Field test

The OF test was designed to measure locomotor activity, explorative behavior, anxiety-like behavior, and head pain/sensitivity (44). The setup consisted of an enclosed square area 1 × 1 m, with anti-reflective walls 34.5 cm high to prevent escape. Testing was performed on day 5 post-surgery. The room was dimly lit to 30 lux to mimic comfortable conditions for the rats. Parameters chosen for analysis of locomotor performance, anxiety-like behavior, exploration and head pain/sensitivity were distance traveled (meters), time spent in the central zone (seconds), number of rearing episodes and time spent head grooming (seconds), respectively. Rats were put in the room at 9:30 a.m. in their cages for acclimatization. Testing was performed between 10 and 12 a.m. Rats were taken out of their cages individually and put in the center of the enclosed area. Once placed, the rat was recorded with a camera and tracked using the ANY-MAZE software while another investigator manually counted rearing episodes as well as head grooming time. Rats were monitored for 10 min. The testing surface was then cleaned with 20% ethanol to remove the scent of previous rats to avoid discrepancies and distractions between tests. The investigator was blinded to the group designation.

#### 2.3.3. Electronical von Frey

Mechanical allodynia is increased in the facial area during migraine attacks, and to a lesser extend in other parts of the body (45). Mechanical allodynia was measured on the rats using an EVF Anesthesiometer (IITC, USA). EVF consist of a filament attached to a sensor of the EVF device. Individual rats were tested on two different occasions, before the actual experiment so rats could get acclimated to the EVF test. Both the periorbital and the plantar surface was chosen for the experiment, to test if CFA affected more than the area it was applied to (periorbital). On the days of testing, rats in their cages were put in the experimental room 30 minutes before testing for acclimatization. Testing was performed between 01 and 04 p.m. on day 4 and 5 post-surgery. To initiate testing, an investigator blinded to the individual rats they were handling, tied the rats firmly with a blanket, to keep the rats steady but still given enough freedom to allow the rats to showcase withdrawal reflexes. For periorbital testing, rats were tied firmly except for their heads which could move freely. The investigator would then apply periorbital pressure with the EVF device until withdrawal reflexes were shown from the rats and the amount of pressure (grams) at the time of withdrawal was recorded by the EVF device. Similarly, for the plantar testing, the investigator tied the rats firmly only allowing hindpaw movement for withdrawal reflexes (46). Both investigators made sure to not surpass pressure above 300 g to not hurt the rats. No response was therefore set to 300 g.

**Figure 2:**
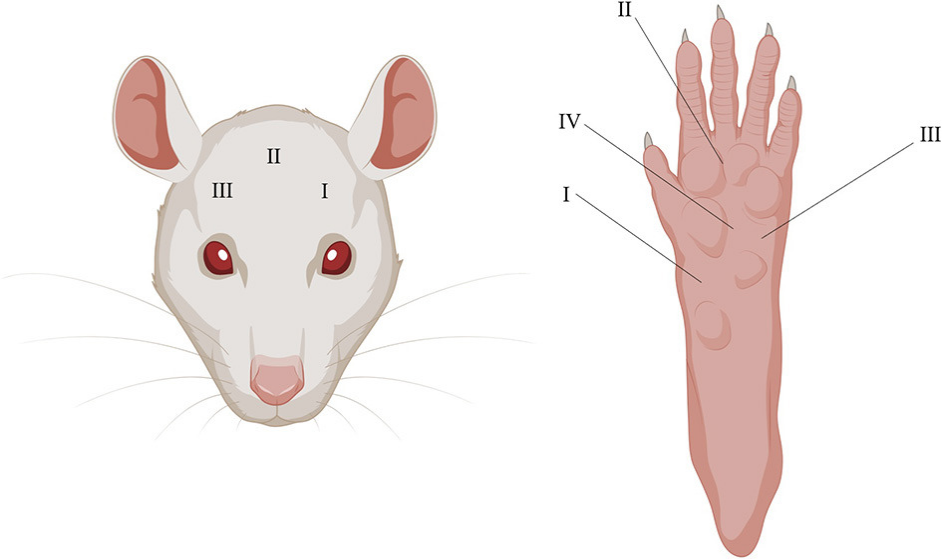
Illustrative depiction of periorbital and plantar testing areas using the EVF. I–III on the (**Left)** and I–IV on the (**Right)** depicts where pressure was applied in chronological order.

Three periorbital areas (I–III) were tested with the EVF device. Location I (the most left area) was tested twice but only the second time was used for the analysis and averaged with the results from the two other locations, as the first time was used as a to habituate the rat to the test. Five total areas (I-IV) were tested with EVF on the right plantar surface and then averaged for analysis (Figure 2).

### 2.4. Immunohistochemistry

On the end of day 5, rats from all the groups were anesthetized with gas (30% CO_2_ in 70% O_2_) and sacrificed by decapitation. TGs were then dissected and incubated in 4% paraformaldehyde in phosphate buffered saline (PBS) for 2–4 h in +4°C. The TGs were then put in 10% sucrose (Merck, Germany) in Sorensen's phosphate buffer (0.1 M NaH_2_PO_4_ and 0.1 M Na_2_HPO_4_) overnight at +4°C. The next day, the tissues were immersed in 25% Sorensen's phosphate buffer at +4°C overnight. On the following day, the TGs were embedded in a gelatin medium (30% egg albumin, 3% gelatin, Merck, Germany), frozen in −20°C and cryosectioned at 10 μm (Leica CM3050 S, Leica Biosystems). The sections were mounted on microscope slides (Hounisen, Germany) and stored at −20°C until use.

The TG sections were thawed at room temperature and then rehydrated and permeabilized with 0.25% Triton X-100 diluted in PBS (PBS-T; Sigma) for 3 × 5 min. The TGs were then blocked for 20 min, washed with a PBS-T containing 1% bovine serum albumin (BSA; Sigma) for 5 min. The TG sections were then incubated with a 1:200 dilution of a primary antibody for CGRP (rabbit, anti-CGRP (D5R8F), Cell Signaling Technology) overnight at +4°C. The following day, the TG sections were washed of excess antibodies in PBS-T with 1% BSA for 3 × 5 min, followed by an incubation with a 1:400 dilution of the secondary antibody (Goat Anti-Rabbit IgG H&L, Alexa Fluor 488 (ab150077), Abcam) diluted in PBS-T for 1 h in a dark room. Excess secondary antibody was thereafter washed off with PBS-T for 3 × 5 min. PBS crystals were washed off with ultrapure water for 1 min. Cover glass was then mounted with anti-fading medium (Vectashield, Vector Laboratories, Burlingame CA, USA) containing 4′,6-diamidino-2-phenylindole (DAPI). Pictures were acquired on a Nikon Ti2-E microscope.

### 2.5. Statistical analysis

We chose a 2:1 setup of sham to CFA to increase the sample size for the inflammation group, as we expected possible larger variations, and to enable a normality analysis of the CFA responses. The responses in the CFA group were normally distributed for all parameters tested using a D'Agostino & Pearson test, the best test for *n* > 10. Statistical analysis was further performed using GraphPad Prism version 8. One-way analysis of variance (ANOVA) was used for comparison between all groups for behavior tests, followed by Tukey's posttest. The ROUT method was used to identify outliers. *P* < 0.05 was considered significant. Data was calculated as mean ± SEM. *n* = number of rats.

## 3. Results

### 3.1. Light dark box (LDB) test

#### 3.1.1. Light zone time

The LDB test can potentially capture photophobic behavior as one of the boxes is significantly more illuminated than the other. More time spent in the light zone could indicate this behavior.

**Figure 3:**
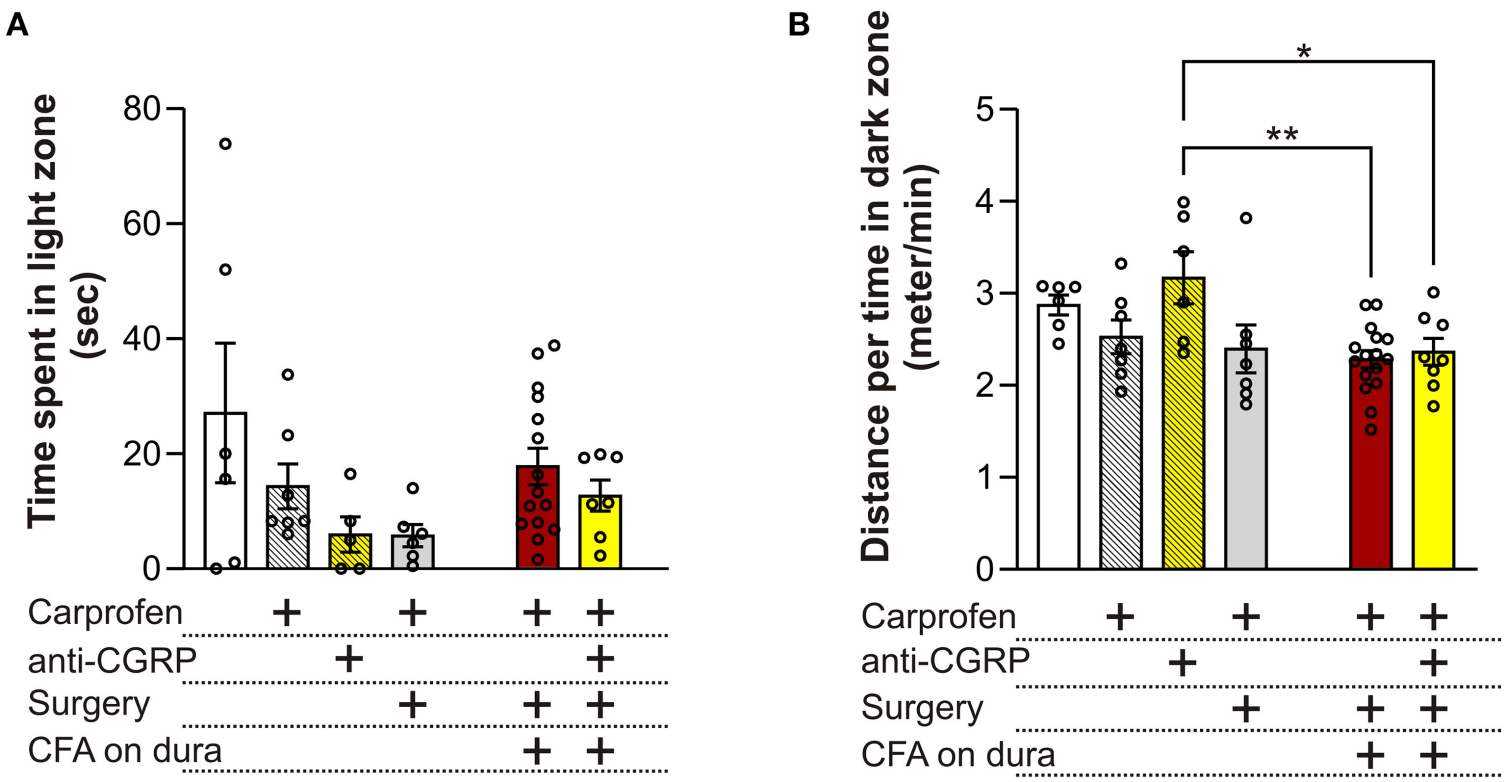
Behavioral results from the LDB test **(A)** No significant differences on time spent in the light zone were observed between the groups. **(B)** No significant differences on the distance traveled during time spent per minute in the dark zone were observed between fresh + carprofen rats, fresh + anti-CGRP rats nor sham rats when compared with fresh rats. No significant difference was observed between sham rats and fresh + carprofen rats. No significant differences on the distance traveled during time spent in dark zone were observed between fresh rats, fresh + carprofen nor sham rats when compared with CFA rats. No significant differences were observed on the distance traveled during time spent in the dark zone between fresh rats nor fresh + carprofen rats when compared with CFA + anti-CGRP rats. CFA + anti-CGRP rats traveled significantly more during their time in the dark zone than fresh + anti-CGRP rats. No significant difference was observed between sham rats and CFA + anti-CGRP rats. Data are shown as mean ± SEM, and *p* values (*p < 0.05, **p < 0.01) from the One-Way ANOVA and Tukey post-tests are depicted in the graphs.

No significant differences in time spent in the light zone were observed between the different groups (*p* = 0.09, Figure 3A), indicating no effect of carprofen, fremanezumab, the surgery nor dural CFA on photophobia. One single outlier was identified and removed in each of these following groups: fresh + anti-CGRP, sham, CFA and CFA + anti-CGRP.

#### 3.1.2. Distance traveled during the time spent in dark zone

Migraineurs with photophobia seek darker areas to minimize the pain and discomfort felt during the ictal phase of a migraine attack. In addition, migraineurs often reduce the amount of physical activity, as movement during a migraine attack can lead to more pain. Therefore, we chose distance traveled per minute spent in the dark zone, as a parameter that could best represent this phenomenon (Figure 3B). Compared to fresh rats (2.9 ± 0.1 min/s) no significant differences in distance traveled during time spent in dark zone were observed when compared to fresh + carprofen rats (2.5 ± 0.2 min/s, *p* = 0.8), fresh + anti-CGRP rats (3.2 ± 0.3 min/s, *p* = 0.9), nor sham rats (2.4 ± 0.3 min/s, *p* = 0.5). These data indicate no apparent effect of carprofen, fremanezumab nor the surgery on the distance traveled during the time they spend in the dark box. Also, no significant difference was observed between fresh + carprofen rats and sham rats (2.5 ± 0.2 min/s vs. 2.4 ± 0.3 min/sec, *p* = 0.9).

No significant differences in distance traveled during time spent in dark zone were observed between CFA rats (2.3 ± 0.1 min/s) and fresh rats (2.9 ± 0.1 min/s, *p* = 0.1), fresh + carprofen rats (2.5 ± 0.2 min/s, *p* = 0.9) nor sham rats (2.4 ± 0.3 min/s, *p* = 0.9). Therefore, dural CFA seem to not have had an effect on this parameter.

Further CFA + anti-CGRP rats showed no significant difference in distance traveled during time spent in dark zone when compared with CFA rats (2.4 ± 0.1 min/s vs. 2.3 ± 0.1 min/s, *p* = 0.9), nor when being compared to fresh rats (*p* = 0.4) or fresh + carprofen rats (*p* = 0.9). However, a significant difference was observed when comparing CFA + anti-CGRP rats with fresh + anti-CGRP rats (2.4 ± 0.1 min/s vs. 3.2 ± 0.3 min/s, *p* = 0.04). No significant difference was observed when comparing CFA + anti-CGRP rats with sham rats (*p* = 0.9). None of the groups spend less time traveling in the dark box than our control rats (fresh + carprofen).

### 3.2. Open Field (OF) test

#### 3.2.1. Distance traveled

**Figure 4:**
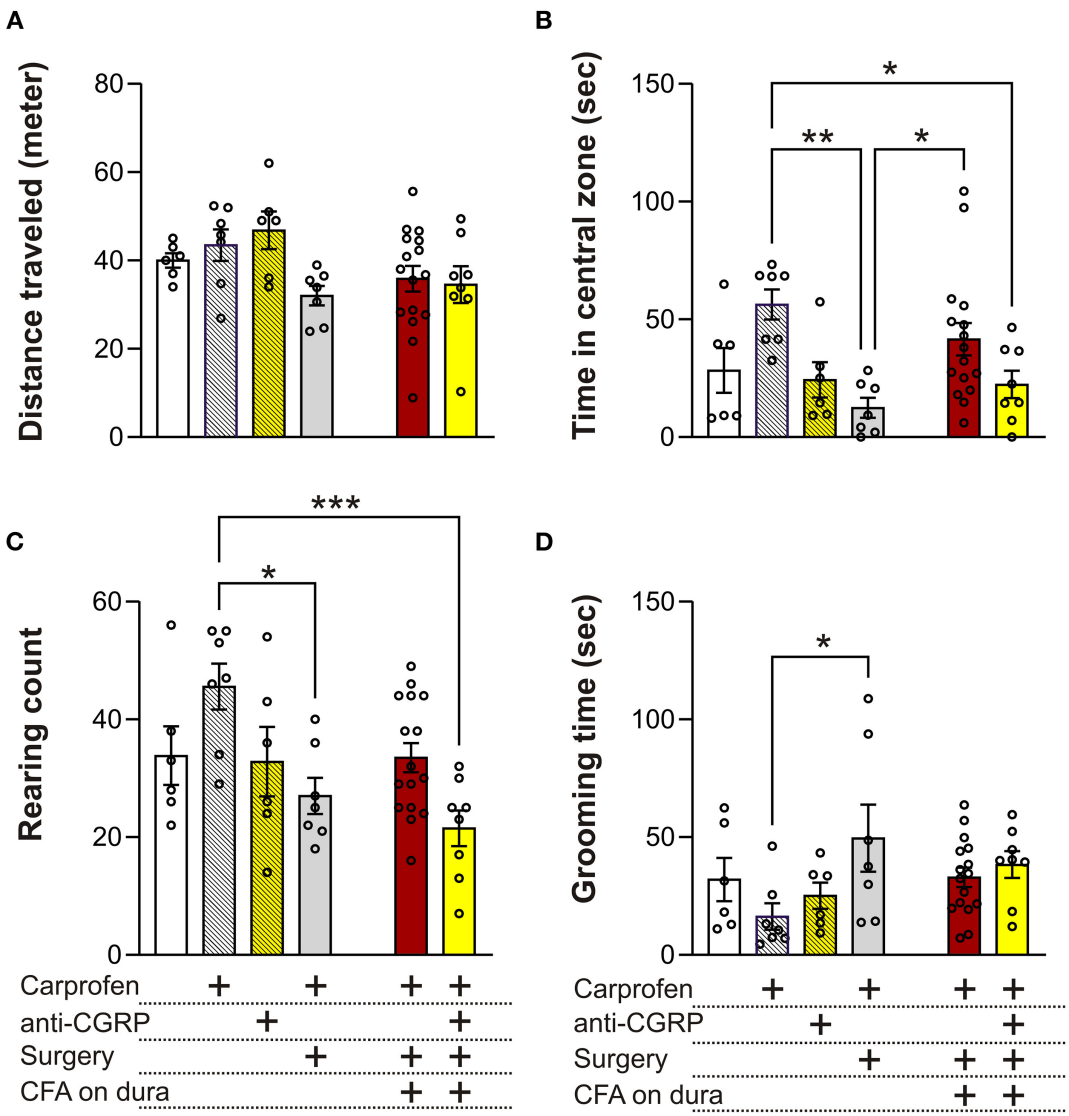
Behavioral results from the OF test. **(A)** No significant differences on the distance traveled were observed between the groups. **(B)** No significant differences in time spent in central zone were observed between fresh + carprofen rats, fresh + anti-CGRP rats nor sham rats when compared with fresh rats. Sham rats spent significantly less time in the central zone than fresh + carprofen rats. No significant differences were observed in time spent in the central zone between fresh rats and fresh + carprofen when compared with CFA rats, but CFA rats spent significantly more time in the central zone than sham rats. No significant differences in time spent in the central zone were observed between CFA rats and fresh rats when compared with CFA + anti-CGRP rats. CFA + anti-CGRP rats spent significantly less time in the central zone than fresh + carprofen rats. No significant difference was observed between CFA + anti-CGRP rats and sham rats. **(C)** No significant difference in rearing count was observed between fresh + carprofen rats, fresh + anti-CGRP rats nor sham rats when compared with fresh rats. sham rats reared significantly less than fresh + carprofen rats. No significant differences in rearing count were observed between fresh rats, fresh + carprofen rats nor sham rats when compared with CFA rats. No significant differences in rearing count were observed between CFA rats nor fresh rats when compared with CFA + anti-CGRP rats. CFA + anti-CGRP rats reared significantly less than fresh + carprofen rats, while no significant differences were observed between fresh + anti-CGRP rats nor sham rats when compared with CFA + anti-CGRP rats. **(D)** No significant differences in grooming time were observed between fresh + carprofen rats, fresh + anti-CGRP rats nor sham rats when compared with fresh rats. sham rats groomed significantly less than fresh + carprofen rats. No significant differences in grooming time were observed between fresh rats, fresh + carprofen rats nor sham rats when compared with CFA rats. No significant differences in grooming time were observed between CFA rats, fresh rats, fresh + carprofen rats, fresh + anti-CGRP rats nor sham when compared with CFA + anti-CGRP rats. Data are shown as mean ± SEM, and *p* values (*p < 0.05, **p < 0.01, ***p < 0.001) from the One-Way ANOVA and Tukey post-tests are depicted in the graphs.

The OF test can allow to measure migraine relevant behaviors like anxiety/depression, exploration, decreased physical activity and head pain. Distance traveled in the OF test is indicative of the locomotor performance of the animals. No significant differences were observed between groups, indicating no effect of carprofen, fremanezumab, the surgery, nor CFA on locomotor activity (Figure 4A).

#### 3.2.2. Central zone time

Less time spent in the central zone in the OF test can be indicative of anxiety- or depression-like behavior. No significant differences in time spend in central zone were observed between fresh (28.3 ± 9.5 s) and fresh + carprofen (56.3 ± 6.4 s, *p* = 0.2), fresh + anti-CGRP (24.3 ± 7.4 s *p* = 0.9), nor sham rats (12.43 ± 4.3 s, *p* = 0.8). These data indicate no apparent effect of carprofen, fremanezumab nor the surgery on anxiety levels. Also, sham rats spend significantly less time in the central zone than fresh + carprofen rats (56.3 ± 6.4 s vs. 12.4 ± 4.3 s, p < 0.01).

No significant difference was observed between CFA rats and fresh rats on time spent in the central zone (41.6 ± 6.9 s vs. 28.3 ± 9.5 s, *p* = 0.8), while significantly more time in the central zone was spent by the CFA rats than sham rats (41.6 ± 6.9 s vs. 12.4 ± 4.3 s, *p* = 0.05). It therefore appears that CFA does not increase anxiety beyond the surgical procedure, on the contrary it indicates that CFA application could reduce anxiety.

CFA + anti-CGRP rats showed no significant difference in central zone time when compared with CFA rats (22.4 ± 5.8 s vs. 41.6 ± 6.9 s, *p* = 0.3) nor fresh rats (*p* = 0.9). However, CFA + anti-CGRP rats spent significantly less time in the central zone than fresh + carprofen rats (22.4 ± 5.8 s vs. 56.3 ± 6.4 s, *p* = 0.04). No significances in central zone time were observed when compared with fresh + anti-CGRP rats (*p* = 0.9) nor sham rats (*p* = 0.9).

#### 3.2.3. Rearing count

A decreased rearing count could indicate lower willingness of animal to explore their surroundings.

No significant differences in rearing count were observed between fresh (33.8 ± 5) and fresh + carprofen rats (45.6 ± 3.9, *p* = 0.3), fresh + anti-CGRP rats (32.8 ± 5.9, *p* = 0.9), nor sham rats (27 ± 3.1, *p* = 0.8), indicating no effect of carprofen, fremanezumab nor the surgery on exploration behavior. Sham rats, however, reared significantly less than fresh + carprofen rats (27 ± 3.1 vs. 45.6 ± 3.9, *p* = 0.02), indicating that the surgery worsened exploration behavior.

No significant differences in rearing count were observed between CFA rats (33.5 ± 2.5) and fresh rats (33.8 ± 5, *p* = 0.9), fresh + carprofen rats (45.6 ± 3.9, *p* = 0.1), nor sham rats (27 ± 3.1, *p* = 0.7). CFA therefore do not appear to have had an impact on exploration behavior.

No significant differences in rearing count were observed between CFA + anti-CGRP rats (21.5 ± 3) and CFA (33.5 ± 2.5, *p* = 0.1) or fresh rats (33.8 ± 5, *p* = 0.3). However, CFA + anti-CGRP rats (21.5 ± 3 g) reared significantly less than fresh + carprofen rats (45.6 ± 3.9, p < 0.001). No significant in rearing differences were observed when comparing CFA + anti-CGRP rats with fresh + anti-CGRP rats (*p* = 0.4) and sham rats (*p* = 0.9).

#### 3.2.4. Grooming time

Rats spending a prolonged amount of time grooming their head could be indicative of head pain (22). No significance differences in grooming time were observed between fresh (32 ± 9.1 s) and fresh + carprofen rats (16.3 ± 5.6 secs *p* = 0.8), fresh + anti-CGRP (25.1 ± 13.6 s, *p* = 0.9), nor sham rats (49.5 ± 14.2 s, *p* = 0.7). However, sham rats groomed significantly more than fresh + carprofen rats (49.5 ± 14.2 s vs. 16.3 ± 5.6 s, p < 0.05).

No significance differences in grooming time were observed between CFA rats (32.9 ± 4.1) and fresh rats (32.0 ± 9.1 s, *p* = 0.9), fresh + carprofen rats (16.3 ± 5.6 s, *p* = 0.5) nor sham rats (49.5 ± 14.2 s, *p* = 0.5).

CFA + anti-CGRP rats (38.4 ± 5.7 s) showed no significant differences in grooming time when compared with CFA rats (32.9 ± 4.1 s, *p* = 0.9), fresh rats (32 ± 9.1 ssec, *p* = 0.9), fresh + carprofen rats (32.9 ± 4.1 s, *p* = 0.3), fresh + anti-CGRP rats (25.1 ± 4.7 s, *p* = 0.8), nor sham rats (49.5 ± 14.2 s, *p* = 0.9). These data indicate that fremanezumab had no alleviating effect.

### 3.3. Electronical von Frey (EVF)

Migraineurs during attacks experience painful headaches and are more sensitive to touch, especially on facial areas, and more than 60% of migraine patients report cutaneous allodynia (47). The EVF test was used to measure mechanical allodynia on the periorbital and plantar surface. The two areas were chosen to assess whether the dural CFA model was specific to the facial area, as this area is more relevant in the context of migraines. The below results are an average of the three measurements (Figure 2), as no differences were observed between any of the individual sites (data not shown).

#### 3.3.1. EVF: Periorbital day 4 and 5

**Figure 5:**
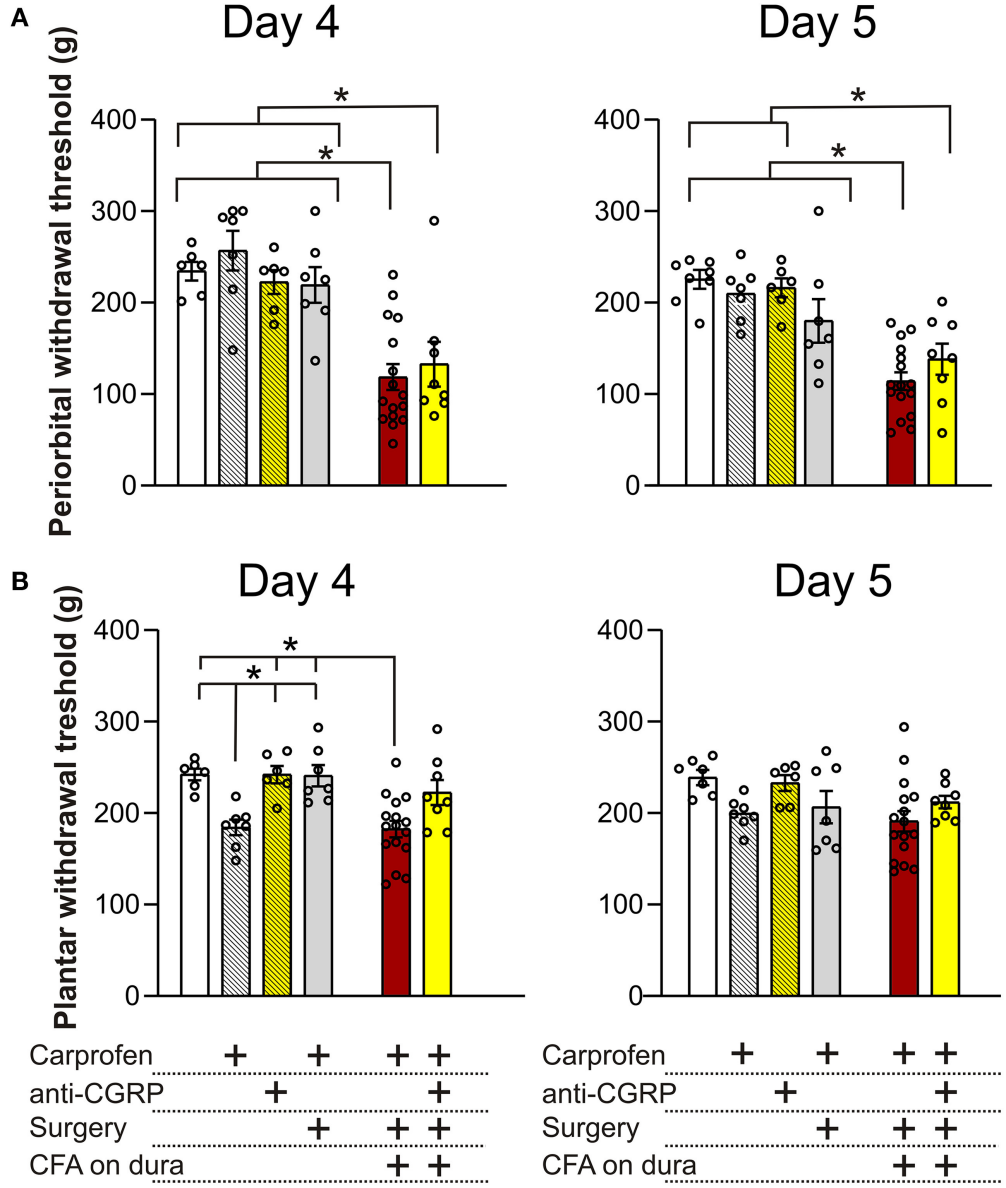
Periorbital and plantar allodynia on day 4 and 5. **(A)** On day 4, no significant differences in periorbital withdrawal thresholds were observed between fresh + carprofen rats, fresh + anti-CGRP rats nor sham rats when compared with fresh rats. CFA rats had significantly lower periorbital withdrawal thresholds than fresh rats, fresh + carprofen rats and sham rats. No significant difference in periorbital withdrawal thresholds was observed between CFA + anti-CGRP and CFA rats, but CFA + anti-CGRP had significant lower periorbital withdrawal thresholds than fresh rats, fresh + carprofen rats, fresh + anti-CGRP rats and sham rats. On day 5, a similar tendency was observed. However, no significant difference in periorbital withdrawal threshold was observed between sham rats and CFA rats. **(B)** On day 4, fresh + carprofen rats had significantly lower plantar withdrawal thresholds than fresh rats. No significant differences were observed between fresh + anti-CGRP rats and sham rats, when compared with fresh rats. Sham rats had significantly higher plantar withdrawal thresholds than fresh + carprofen rats. CFA rats had significantly lower plantar withdrawal thresholds when compared with fresh rats. No significant difference was observed between CFA rats and fresh + carprofen rats. CFA rats had significantly lower plantar withdrawal thresholds than sham rats. None of the groups had significant differences than CFA + anti-CGRP rats. On day 5, no differences in plantar withdrawal thresholds were observed between groups. Data are shown with mean ± SEM, and *p* values (at least ^*^p < 0.05 from the One-Way ANOVA and Tukey post-tests are depicted in the graphs).

On day 4 on the periorbital areas (Figure 5A), fresh rats (234.4 ± 10.2 g) showed no significant differences when compared to fresh + carprofen rats (256.9 ± 21.7 g, *p* = 0.9), fresh + anti-CGRP rats (222.6 ± 12.9, *p* = 0.9) and sham rats (219.3 ± 19.5 g, *p* = 0.9). These data indicate no effect of carprofen, fremanezumab nor the surgery on periorbital withdrawal thresholds on day 4. Also, no significant difference in periorbital withdrawal thresholds was observed between fresh + carprofen rats and sham rats (256.9 ± 21.7 g vs. 219.3 ± 19.5 g, *p* = 0.8), indicating no effect of the surgery on periorbital withdrawal thresholds on day 4.

Significantly lower periorbital withdrawal thresholds were observed from CFA rats (118.5 ± 14.2 g) when compared with fresh rats (234.4 ± 10.2 g, *p* < 0.001), fresh + carprofen rats (256.9 ± 21.7 g, *p* < 0.0001) and sham rats (219.3 ± 19.5 g, *p* < 0.01). These data indicate that CFA caused lower periorbital withdrawal thresholds.

CFA + anti-CGRP rats showed no significant difference in periorbital withdrawal thresholds when compared with CFA rats (132.7 ± 24.5 g vs. 118.5 ± 14.2 g, *p* = 0.9), while significant lower periorbital withdrawal thresholds were observed when compared with fresh rats (234.4 ± 10.2 g, *p* = 0.01), fresh + carprofen rats (256.9 ± 21.7 g, *p* = 0.001), fresh + anti-CGRP rats (222.6 ± 13 g, *p* = 0.04) and sham rats (219.3 ± 19.5 g, *p* = 0.03). These data indicate that fremanezumab had no impact on alleviating the effect of CFA on periorbital allodynia on day 4.

A similar tendency was observed on day 5, when comparing fresh rats (225.4 ± 10.3 g) with fresh + carprofen rats (210 ± 11.2 g, *p* = 0.9), fresh + anti-CGRP rats (215.5 ± 10.5 g, *p* = 0.9) and sham rats (180.1 ± 23.9 g, *p* = 0.4). These data indicate no effect of carprofen, fremanezumab nor the surgery on periorbital withdrawal thresholds. Also, no significant difference in periorbital withdrawal thresholds was observed between fresh + carprofen rats and sham rats (210 ± 11.2 g vs. 180.1 ± 23.9 g, *p* = 0.7), indicating no effect of the surgery on periorbital withdrawal thresholds on day 5.

Lower periorbital withdrawal thresholds were observed between CFA rats (114.2 ± 9.6 g) and fresh rats (225.4 ± 10.3 g, *p* < 0.0001), fresh + carprofen rats (210 ± 11.2 g, *p* < 0.0001) and sham rats (180.1 ± 23.9 g, p < 0.01). These data indicate that CFA still was the cause of lower periorbital withdrawal thresholds.

CFA + anti-CGRP rats (138.1 ± 17.1 g) had no significant difference in periorbital withdrawal thresholds when compared with CFA rats (114.2 ± 9.6 g, *p* = 0.8), but showed significantly lower periorbital withdrawal thresholds than fresh rats (225.4 ± 10.3 g, p < 0.01), fresh + carprofen rats (210 ± 11.2 g, p < 0.01), fresh + anti-CGRP rats (215.5 ± 10.5 g, *p* = 0.01). Interestingly, no significance was observed when compared with sham rats (120.3 ± 11 g vs. 180.1 ± 23.9 g, *p* = 0.3). These data indicate that fremanezumab had little to no impact on alleviating the effect of CFA on periorbital allodynia on day 5.

#### 3.3.2. EVF: Plantar day 4 and 5

On day 4 on the plantar areas (Figure 5B), fresh rats (242.2 ± 6.4 g) had significantly higher thresholds than fresh + carprofen rats (184.3 ± 8.6 g, *p* = 0.02) but not when compared to fresh + anti-CGRP rats (242.0 ± 9.4 g, *p* = 0.9) or sham rats (240.8 ± 11.6 g, *p* = 0.9). Also, fresh + carprofen rats had significantly lower plantar withdrawal thresholds than sham rats (184.3 ± 8.6 g vs. 240.8 ± 11.6 g, *p* = 0.02).

CFA rats (182.4 ± 9 g) had significantly lower plantar withdrawal thresholds than fresh rats (242.2 ± 6.4 g, *p* < 0.01), but not when compared to fresh + carprofen rats (184.3 ± 8.6 g, *p* = 0.9). Also, CFA rats had significantly lower plantar withdrawal thresholds than sham rats (182.4 ± 9 g vs. 240.8 ± 11.6 g, *p* < 0.01).

No significant differences in plantar withdrawal thresholds were observed between CFA + anti-CGRP rats (222.4 ± 13.7 g) and CFA rats (182.4 ± 9 g, *p* = 0.051), fresh rats (242.2 ± 6.4 g, *p* = 0.8), fresh + carprofen rats (184.3 ± 8.6 g, *p* = 0.2), fresh + anti-CGRP rats (242 ± 9.4 g, *p* = 0.8) nor sham rats (240.8 ± 11.6 g, *p* = 0.9). None of the groups were significantly more sensitive than our control rats (fresh + carprofen), suggesting that the plantar region is not sensitized.

On day 5 on the plantar region, no significant differences were observed between any of the groups, indicating no effect of the surgery nor CFA on plantar withdrawal thresholds at this time point, suggesting that day 5 is the optimal day for concluding on the periorbital allodynia.

### 3.4. Immunohistochemistry (IHC)

**Figure 6:**
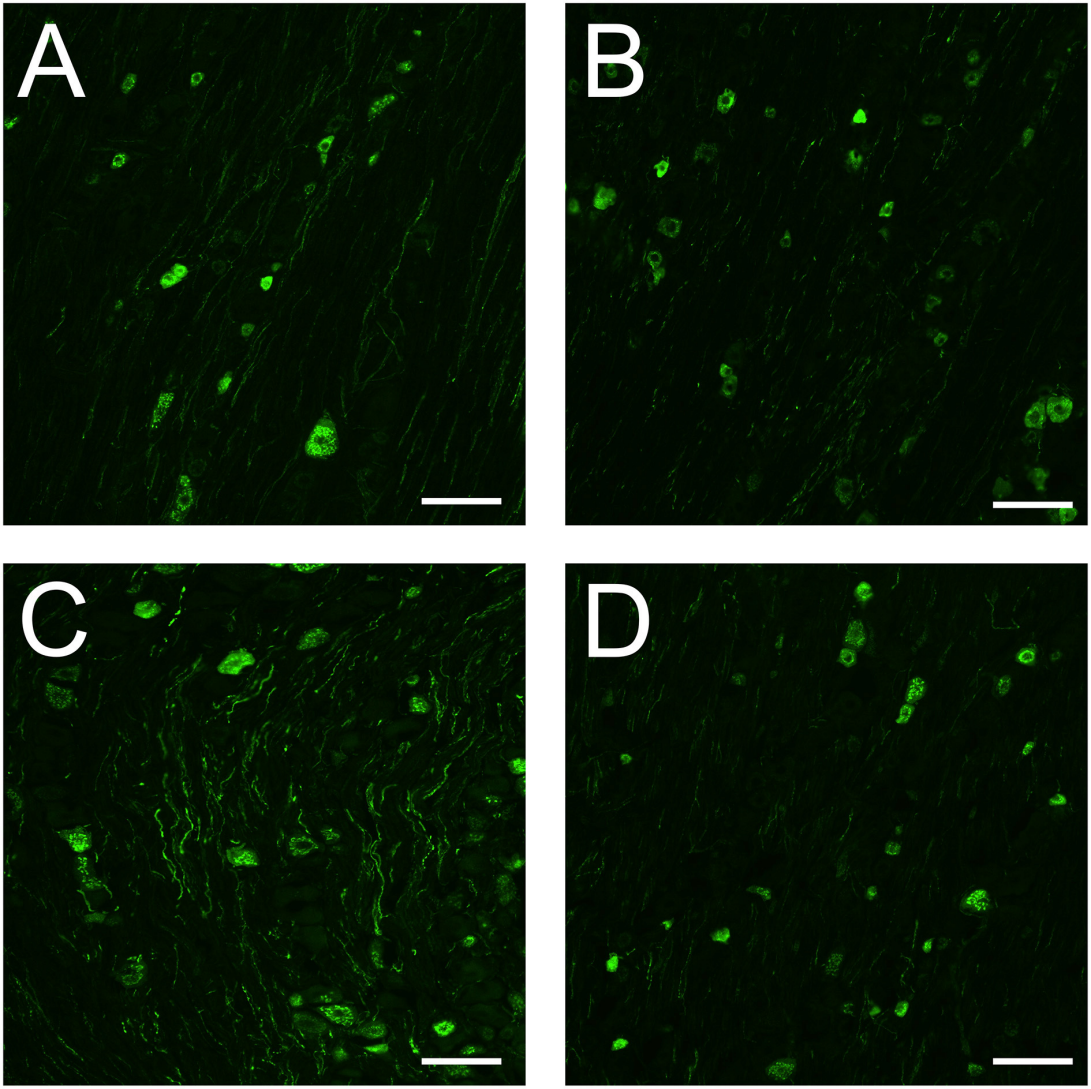
Immunohistochemistry on CGRP in the TG. **(A)** In the TG of fresh rats, granular shaped patterns were detected in the cytoplasm of CGRP positive neurons, while CGRP positive fibers were button shaped. **(B)** In the TG of sham rats, similar patterns were detected in both neurons and fibers to that of fresh rats. The immunoreactivity of both neurons and fibers appears similar in intensity to that of fresh rats. **(C)** In the TG of CFA rats, similar patterns were detected in both neurons and fibers to that of fresh and sham rats. The immunoreactivity of CGRP positive neurons were like that of fresh and sham rats, while the immunoreactivity of the fibers appears more enhanced. **(D)** In the TG of CFA + anti-CGRP rats, similar patterns were observed as in previous groups. The immunoreactivity of both neurons and fibers appears similar in intensity to that of fresh and sham rats. Scale bar is 100 μmeter.

We previously found that dural CFA leads to stronger immunoreactivity of CGRP positive neurons and nerve fibers when compared to fresh animals. In this study, fresh rats had CGRP immunoreactivity in some neurons and nerve fibers at day 5 (Figure 6A). In the neurons, the immunoreactivity is localized in the cytoplasm in granular shapes around the nucleus, potentially staining CGRP in large dense-core vesicles. In the nerve fibers, CGRP can be found stained in thin and bouton shaped fibers, that most likely are C-fibers. A same pattern can be observed in sham rats (Figure 6B), indicating that the surgery most likely did not affect CGRP in the TG. CFA rats also have immunoreactivity in neurons and nerve fibers. However, the immunoreactivity appears stronger when compared with fresh rats and sham rats at day 5, at least in the fibers (Figure 6C). This is in line with our previous study. Interestingly, the immunoreactivity of CGRP in the CFA + anti-CGRP animals appear to resemble a pattern that is closer to that of fresh and sham animals (Figure 6D). This indicates that IV fremanezumab could reduce the increased CGRP caused by CFA in the TG.

## 4. Discussion

In the current study we investigated the behavior outcome following a dural stimulation with CFA and found that CFA induced periorbital allodynia. This allodynia is specific to the periorbital area but could not be inhibited by an anti-CGRP antibody such as fremanezumab. Below we discuss this in the light of the current view of migraine pathophysiology.

Sterile neurogenic inflammation of the dural meninges has been suggested to play a part in migraine pathology since the 1980s (48). There is a growing amount of evidence that neurogenic inflammation could be a key element in the sensitization process underlying migraine, with particular relevance to chronification (49–52). Our line of investigation started by studying inflammatory pathways in primary TG neurons isolated from rats (53, 54). This was followed by the administration of CFA onto the dura (20), which was inspired by the TG activation following CFA injection in the temporomandibular joint (55). However, behavior outcomes have never been tested in this model.

The LDB experiments are linked to the common symptom of migraine in humans, namely photophobia (56). The mechanism behind photophobia is unknown but it has been suggested that convergence of optical signals from retinal photoreceptors and nociceptive signals from the TGVS, could play a role (43). Although the LDB has been used in migraine research (57), one must keep in mind that rodents naturally show light aversive behavior (58). In the current paper we have chosen to focus on two outcomes, which was time spent in light zone and distance covered in the dark zone per minute (Figure 3). We did not observe any differences between our groups using this approach, and in general the animals spent very little time in the light zone. General anxiety is not likely associated with CFA injections, and no differences were observed in the LDB in studies following intraplantar or intraperitoneal injections of CFA (59, 60). This contrasts to other migraine models such as NTG injections where light aversive behavior has been reported (61).

In addition to feeling migraine relief by avoiding light, many migraineurs also feel that the pain is exacerbated by physical activity (62), which is also stated in the diagnostic criteria for migraine (63). This could in part be mimicked in an OF test, but as for the LDB, this test also includes an element of anxiety (64). The data in the current study did not show any differences related to the distance traveled, following the application of CFA. However, there was a difference in time spent in the central zone, where animals from the sham group, avoided the central zone, and CFA application to the dura mitigated this behavior (Figure 4). In other inflammation models e.g., where IS was applied on the dura, increased rest and decreased exploratory behavior was reported (39). However, this contrast to the data by Liu and colleagues (65), where IS rats traveled more and spent more time in the central zone during OF testing. Interestingly, in their study anti-CGRP treatment mitigated this behavior (65). It could be possible that CFA triggers some anti-anxiety mechanisms which are currently unknown.

Spontaneous rearing behavior has been shown to have a strong relationship to the hippocampus (66). Although not linked directly to migraine pathology, the hippocampus is a key target of stress response, and hippocampus-dependent behaviors are strongly influenced by stress (67). A study which chronically administered IS to the dura of rats, found decreased rearing behaviors in rats (67). In our study, we observed that rearing was lower in the sham animals when compared to the fresh + carprofen rats, we believe that this change in behavior is linked to the surgical procedure itself. Similarly, for grooming, there was an increase in the sham animals compared to fresh + carprofen animals and it is therefore probably also linked to the surgical procedure. Therefore, for the sham group it is likely that the mechanical stimulation of the dura mater might activate some pain pathways (34), which could be considered mechanical stimulus and then the additional CFA as the chemical stimulus group. Other studies (using IS) have shown increased face rubbing (68) and grooming (69), but in our hands we cannot discriminate the increased grooming behavior between sham and CFA, suggesting either that sham surgery activates TGVS or that the incision itself leads to grooming. The CFA approach include a craniotomy and it has been shown in other work that the mere attachment of the rat in the stereotaxic frame can activate the trigeminal system (70). This highlights importance of using both fresh and sham rats to uncover the true role of inflammation and separate it from the invasive measures (71).

Some migraine provoking agents such as CGRP and PACAP have been shown to induce periorbital allodynia following subcutaneous periorbital injections (72). Furthermore, when IS was applied to the dura it can cause periorbital and/or plantar allodynia (37, 73). In the current study we observed that CFA caused periorbital allodynia, which was not observed in the sham animals. Importantly, the CFA induced allodynia is specific to the periorbital area, as no allodynia is observed in the plantar region. This differs from several other models of migraine, where both periorbital and plantar sensitivity is increased (73–76). Although activation of the trigeminal nucleus caudalis (TNC) and central sensitization does occur following CFA-induced activation of the dura mater (77), the central sensitization cannot be as widespread, compared to what is observed for other models.

In the current study, we did not see a mitigating effect of the anti-CGRP antibody. This contrasts to some previous data as it has been shown that α-CGRP_(8 − 37)_, a CGRP receptor antagonist, was able to reduce allodynia induced by intraplantar CFA (78). Further, the gepant BIBN4096BS (olcegepant) applied topically or intravenously (32) was also able to alleviate inflammatory pain from subcutaneous administered CFA in the plantar region. Both approaches target the CGRP receptor, which contrasts to targeting CGRP itself, using an anti-CGRP antibody. In the current study we cannot exclude that the concentration of antibody is possibly not high enough, as the antibody is competing with CGRP receptors for the binding of CGRP and therefore a large increase in CGRP could overpower the antibody locally. Furthermore, these molecules are very effective at alleviating migraine symptoms in about half of the patients, indicating that not all migraines are linked to CGRP (79). Alternatively, CGRP may not have a significant role in causing allodynia, at least when in presence of other potential pathological markers that CFA may have caused to be upregulated or activated. We further investigated CGRP expression using IHC, and it appears that CFA rats had C-fibers that were loaded more with CGRP in the TG when compared to both fresh and sham rats (Figure 6), indicating enhanced presence of CGRP after CFA application to the dura. This is in line with our previous findings (20). Adding to our previous work, it appears that there is visually less CGRP in the IHC following anti-CGRP intervention, in the CFA + anti-CGRP rats (Figure 6). There is one caveat in these findings, as the anti-CGRP treatment itself could hide the CGRP epitope in the animals.

Although not optimal in studying pain and inflammation, we in the current study, for the wellbeing of the animals, added two doses of painkillers (carprofen) as this was requested during the ethical approval procedure. To control for this we included two groups, fresh and fresh + carprofen to investigate any confounding effects. Although it in the current setup is impossible to fully conclude if carprofen had any mitigating effect following surgery, the *per se* effects appear minimal. We applied a broad approach of behaviors outcomes that is linked to migraine pathology. Nevertheless, we can never be sure that the rodents experience migraine pain, and both the OF test and LDB test carries elements of anxiety in their outcomes. The strength in our study is therefore the specific orofacial allodynia, which is not seen in other models, which could therefore be a new model to understand local vs. systemic/central sensitization which is observed in other migraine models. In line with this, additional molecular outcomes could be studied in future rodent studies with anti-CGRP treatment, such as activation of c-Fos in the TNC (77).

## 5. Conclusion

The current data show that CFA applied to the dura specifically induces periorbital allodynia. However, this is not followed by any other migraine related symptomatic behavior in our model. Where does this leave us with CFA as a model with elements of chronification in migraine? Several previous papers have applied IS rather than CFA, which consist of endogenous inflammatory mediators (histamine, bradykinin, PGE_2_ and serotonin, and they are usually mixed in acidic PBS). Meanwhile, CFA is a water/oil emulsion consisting of dried and inactivated mycobacteria. We believe that CFA could generate local inflammatory markers, without direct receptor induced activation. In addition, IS could lead to indirect release of other signaling molecules such as ATP (80, 81), complicating the interpretation as ATP is involved in migraine pathology (82, 83) [discussed in detail elsewhere (20)]. Indeed, the CFA approach appears to be more specifically localized to the periorbital region and does not induce widespread allodynia. However, in certain IS approaches, plantar allodynia appears only after chronic administration. Therefore, we cannot exclude that CFA would eventually lead to plantar allodynia if applied chronically. Nonetheless, periorbital allodynia often first appears after chronic use in the IS models (37), while from our model, CFA causes periorbital allodynia after single administration, even five days later.

Combining the current data with our previous results, we have collectively shown that the application of CFA onto the dural surface activates the TG (20, 84–86) and that it leads to increased expression of c-Fos, of the central part of the TGVS (77). This activation and molecular changes lead to CFA related outcomes in the rats, where specific periorbital allodynia is a hallmark of this model. In our hands, treating the rats with an IV injection of anti-CGRP antibodies could not significantly mitigate the allodynia. Further work is needed to investigate whether CFA induced allodynia has pathological elements that are not CGRP dependent or whether the CGRP levels are increased by such an extent that the anti-CGRP antibody is unable to significantly reduce the allodynia.

## Data availability statement

The original contributions presented in the study are included in the article/supplementary material, further inquiries can be directed to the corresponding author.

## Ethics statement

The animal study was reviewed and approved by Danish Animal Inspectorate (Dyreforsøgstilsynet).

## Author contributions

PR: conceptualization, methodology, investigation, formal analysis, visualization, and writing—original draft preparation. JB: investigation, methodology, data curation, and writing—reviewing and editing. LE: conceptualization, resources, and writing—reviewing and editing. KH: conceptualization, resources, methodology, investigation, formal analysis, visualization, supervision, project administration, and writing— original draft preparation. All authors contributed to the article and approved the submitted version.
